# Peptide Array on Cellulose Support—A Screening Tool to Identify Peptides with Dipeptidyl-Peptidase IV Inhibitory Activity within the Sequence of α-Lactalbumin

**DOI:** 10.3390/ijms151120846

**Published:** 2014-11-13

**Authors:** Isabelle M. E. Lacroix, Eunice C. Y. Li-Chan

**Affiliations:** Faculty of Land and Food Systems, Food, Nutrition and Health Program, the University of British Columbia, 2205 East Mall, Vancouver, BC V6T 1Z4, Canada; E-Mail: isablac@mail.ubc.ca

**Keywords:** peptide array, dipeptidyl-peptidase IV (DPP-IV) inhibitors, α-lactalbumin, peptide–DPP-IV interaction

## Abstract

The inhibition of the enzyme dipeptidyl-peptidase IV (DPP-IV) is an effective pharmacotherapeutic approach for the management of type 2 diabetes. Recent findings have suggested that dietary proteins, including bovine α-lactalbumin, could be precursors of peptides able to inhibit DPP-IV. However, information on the location of active peptide sequences within the proteins is far from being comprehensive. Moreover, the traditional approach to identify bioactive peptides from foods can be tedious and long. Therefore, the objective of this study was to use peptide arrays to screen α-lactalbumin-derived peptides for their interaction with DPP-IV. Deca-peptides spanning the entire α-lactalbumin sequence, with a frame shift of 1 amino acid between successive sequences, were synthesized on cellulose membranes using “SPOT” technology, and their binding to and inhibition of DPP-IV was studied. Among the 114 α-lactalbumin-derived decamers investigated, the peptides ^60^WCKDDQNPHS^69^ (α*K*_i_ = 76 µM), ^105^LAHKALCSEK^114^ (*K*_i_ = 217 µM) and ^110^LCSEKLDQWL^119^ (*K*_i_ = 217 µM) were among the strongest DPP-IV inhibitors. While the SPOT- and traditionally-synthesized peptides showed consistent trends in DPP-IV inhibitory activity, the cellulose-bound peptides’ binding behavior was not correlated to their ability to inhibit the enzyme. This research showed, for the first time, that peptide arrays are useful screening tools to identify DPP-IV inhibitory peptides from dietary proteins.

## 1. Introduction

Dietary proteins are known to be instrumental in a wide range of nutritional and biological processes [1]. Over the last two decades, a growing body of research has shown that they can be precursors of an array of biologically active peptides that have the potential to improve human health [2]. Even though bioactive peptides have been found encrypted in the sequence of a number of proteins from both plant and animal sources, milk proteins are currently considered the most important precursors of peptides with biological activities [3]. In addition to containing protein fragments with anti-hypertensive, anti-bacterial, anti-cariogenic, anti-oxidative, mineral binding, opioid and immunomodulating activities [4,5,6], dietary proteins, particularly whey proteins, have recently been found to also contain within their sequence peptides able to inhibit the activity of the enzyme dipeptidyl-peptidase IV (DPP-IV) *in vitro* [7,8,9,10,11,12,13,14,15]. The DPP-IV enzyme is known to inactivate the incretins glucose-dependent insulinotropic polypeptide (GIP) and glucagon-like peptide-1 (GLP-1), two gut derived-hormones that play crucial roles in glucose regulation by stimulating pancreatic glucose-dependent insulin, suppressing glucagon release, promoting β-cell proliferation and survival, retarding gastric emptying and modulating appetite [16,17]. Prolonging the half-lives of the incretin hormones by administration of orally available DPP-IV inhibitors such as the peptidometic compounds sitagliptin, vildagliptin and saxagliptin, is currently a promising strategy for the management of type 2 diabetes [18]. Although peptides derived from dietary proteins have not yet been shown to prevent the degradation of the incretins *in vivo*, the discovery of their ability to inhibit the activity of the DPP-IV enzyme *in vitro* has triggered great interest in the bioactive peptide research area.

The traditional approach to study bioactive peptides from dietary proteins typically involves a number of steps, such as hydrolysis of the proteins by enzymatic treatment, isolation of the active peptides, identification of the peptides’ amino acid sequence and finally chemical synthesis of the identified peptides for validation of their biological activity [19,20]. This methodology has recently been used to identify peptides with DPP-IV inhibitory activity from casein [10], whey [15], fish [21,22] and rice bran [23] proteins. However, this empirical way of studying bioactive peptides is rather tedious and presents a number of limitations. It is technically nearly impossible to characterize all bioactive peptides present within a protein hydrolysate and only those that are released from the parent protein during the enzymatic treatment can be identified by this approach. Another investigation strategy that has been successfully used to identify bioactive peptides consists of chemically synthesizing amino acid fragments found within dietary proteins based on their structural properties and similarities with peptides previously reported to have known activities [19]. Yet, synthesizing and screening a large number of peptides using the traditional methods for peptide synthesis can be expensive and time consuming, thus limiting the applicability of this approach [24].

First introduced more than two decades ago, peptide array technology has been developed as a complementary method to the traditional solid phase peptide synthesis to allow the parallel production of hundreds to thousands of peptides [24]. Cellulose-bound peptide arrays, which are cellulose membranes on which small amounts of peptides are built, have been used as screening tools for a wide range of applications, including the study of peptide-antibody, peptide-receptor, peptide-metal ion and peptide-enzyme interactions. In addition, peptide arrays can also be utilized in assays requiring soluble peptides by cleaving them off the membrane [24,25,26]. Despite the numerous possible applications of peptide arrays, to our knowledge, this approach has never been used to identify bioactive peptides, such as DPP-IV inhibitors, from dietary proteins.

The objective of this study was to evaluate the potential of peptide arrays to serve as screening tools to identify DPP-IV inhibitory peptides. Using “SPOT” technology, deca-peptides spanning the entire sequence of α-lactalbumin, a protein previously found to contain within its primary sequence fragments able to inhibit the activity of DPP-IV [14,15], were synthesized on cellulose membranes and their binding to and inhibition of DPP-IV were investigated.

## 2. Results

### 2.1. Binding of Dipeptidyl-Peptidase IV (DPP-IV) to Deca-Peptides on the Array

**Figure 1:**
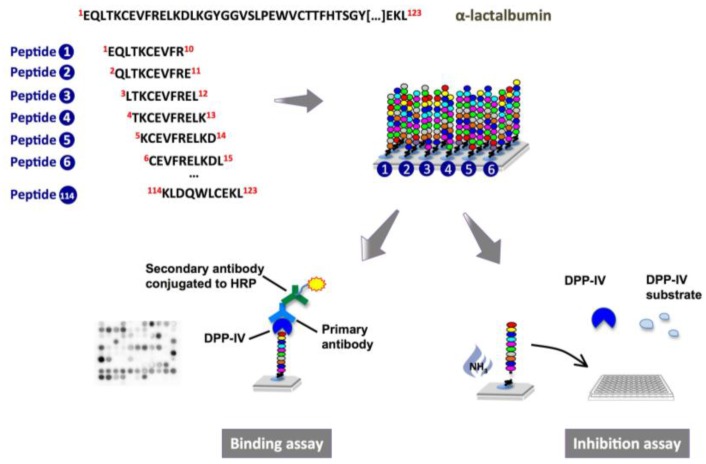
Schematic representation of peptide array synthesis, binding and inhibition experiments. Binding of α-lactalbumin-derived decamers to the dipeptidyl-peptidase IV (DPP-IV) enzyme was investigated directly on the cellulose membrane, whereas the inhibition of DPP-IV was measured using a colorimetric assay with the cleaved peptides.

**Figure 2:**
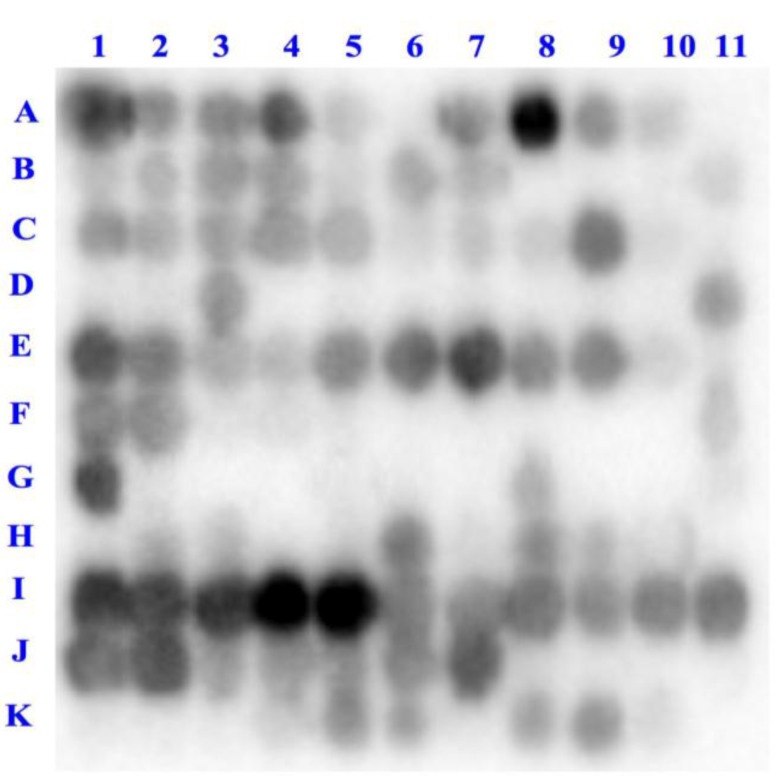
Binding of DPP-IV to deca-peptides derived from α-lactalbumin (A1–K4, Table S1) and from other proteins (K5–K11, Table S1 and Table S2). The binding is inversely correlated to the light signal, the darkest spots representing peptides on which more enzyme was bound.

The interaction between the DPP-IV enzyme and deca-peptides spanning the entire α-lactalbumin sequence (Table S1) was first determined by immunoassay and visualized using an enhanced chemiluminescence substrate (Figure 1). As shown in Figure 2, the probing of the peptide array with DPP-IV revealed that a number of α-lactalbumin-derived peptides are able to interact with the enzyme (dark spots on the array). Since every consecutive spot on the membrane differs by only one amino acid, the presence of consecutive dark spots indicates that some regions of the α-lactalbumin molecule such as ^1^EQLTKCEVFRELK^13^ (spots A1–A4), ^45^NDSTEYGLFQINNKIWCK^62^ (spots E1–E9) and ^89^IMCVKKILDKVGINYWLAHKALCSEKL^115^ (spots I1–J7) were able to bind to DPP-IV while others like ^61^CKDDQNPHSSNICN^74^ (spots F6–F10) and ^68^HSSNICNISCDKFLD^82^ (spots G2–G7) did not seem to interact with the enzyme.

In addition to the 114 α-lactalbumin-derived deca-peptides, seven additional decamers were synthesized on the membrane (spots K5–K11, Table S1 and Table S2). These peptides were selected based on their known effect on DPP-IV activity, their similarity to peptides previously shown to have inhibitory activity or because they are derived from natural substrates for the enzyme (Table S2). Among these, only five were found to bind to DPP-IV, namely the β-lactoglobulin-derived peptides IVTQTMKGLD and LKPTPEGDLE, and the peptides HSQGTFTSDY, YAEGTFISDY and HAEGTFTSDY derived from glucagon, GIP and GLP-1, respectively.

### 2.2. DPP-IV Inhibitory Activity of SPOT-Synthesized Deca-Peptides

In order to determine whether the binding of DPP-IV to the α-lactalbumin-derived peptides is indicative of their ability to inhibit the activity of the enzyme, the cellulose-bound peptides were cleaved off from the membrane and their DPP-IV inhibitory activity was assessed (Figure 1). Among the 114 deca-peptides derived from α-lactalbumin, 33 were able to decrease DPP-IV activity by at least 20% (Table 1). The regions of the protein comprising the amino acids ^84^DLTDDIMCVKKIL^96^ (spots H7–H10), ^97^DKVGINYWLAHKA^109^ (spots I9–J1) and ^105^LAHKALCSEKLDQWLC^120^ (spots J6–K1) appeared to be particularly effective at inhibiting DPP-IV. Amid the seven additional decamers not derived from α-lactalbumin, the sequences IVTQTMKGLD and HSQGTFTSDY, previously found to have some DPP-IV inhibitory activity [15,27] showed 16% and 15% inhibition, respectively. With the exception of GLP-1_(1–10)_, all other non-α-lactalbumin-derived peptides caused more than 23% inhibition of the enzyme’s activity.

**Table 1:** Percent inhibition of DPP-IV activity caused by SPOT-synthesized deca-peptides derived from α-lactalbumin (A1–K4, Table S1) and from other proteins (K5–K11, Table S1 and Table S2). Values equal to or greater than 20% inhibition are highlighted in gray. Percent inhibition values obtained for the decamers not derived from α-lactalbumin are shown in red font. The data are reported as the averages of four determinations obtained from two separate inhibition experiments.

	1	2	3	4	5	6	7	8	9	10	11
**A**	11	16	20	20	17	16	18	14	17	14	8
**B**	11	11	13	13	11	17	15	15	15	15	9
**C**	26	14	18	26	16	10	5	13	12	11	14
**D**	11	9	11	14	14	14	11	15	13	14	11
**E**	14	14	13	9	10	11	9	13	20	6	21
**F**	22	17	19	21	29	15	16	15	15	8	15
**G**	11	9	16	19	25	31	17	19	18	21	22
**H**	19	18	18	21	14	19	25	20	20	20	15
**I**	17	19	26	18	17	20	19	16	22	22	20
**J**	25	18	15	20	16	27	21	20	24	21	25
**K**	22	19	24	19	16	23	25	15	24	19	31

**Figure 3:**
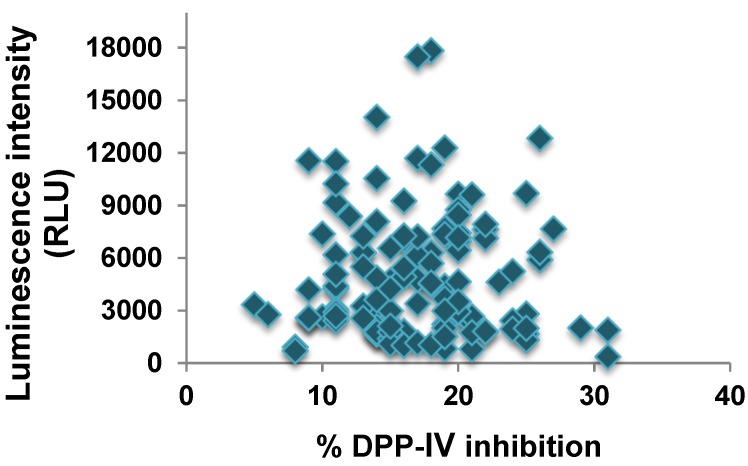
Correlation between binding of DPP-IV to deca-peptides on the array (indicated by luminescence intensity) and inhibition of the enzyme activity by the peptides.

Interestingly, the binding of the deca-peptides to the DPP-IV enzyme was not correlated to their inhibitory activity (Figure 3). In fact, the peptide sequences displaying the strongest binding to DPP-IV, VKKILDKVGI and KKILDKVGIN, caused less than 20% reduction of the enzyme’s activity, whereas the most potent decamers, WCKDDQNPHS, ICNISCDKFL and YPSKPDNPGE, showed moderate or no binding to the enzyme.

### 2.3. Validation of the DPP-IV Inhibitory Activity of the Deca-Peptides

**Figure 4:**
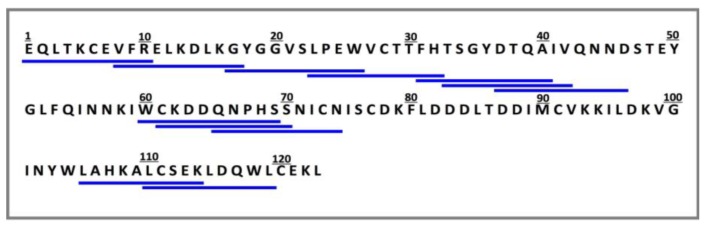
Mature amino acid sequence of α-lactalbumin (UniProt KB database accession number P00711). Deca-peptides underlined were chemically synthesized according to the traditional method of solid phase peptide synthesis to validate their effect on DPP-IV activity.

Being built on the membrane in small amounts, the SPOT-synthesized peptides cannot easily be purified or quantified. Therefore, to validate the DPP-IV inhibitory activity results, a number of deca-peptides found to cause high and low inhibition of DPP-IV activity were obtained by traditional solid phase peptide synthesis and tested for their effect on DPP-IV activity (Figure 4).

The peptides obtained by the two synthesis methods displayed consistent trends in their DPP-IV inhibitory activity (Table 2). The deca-peptides WCKDDQNPHS and IPAVFKIDAL, with 51% and 48% inhibition respectively, were the most potent inhibitors. Conversely, the α-lactalbumin-derived peptides VFRELKDLKG, FHTSGYDTQA, TSGYDTQAIV, DTQAIVQNND, CKDDQNPHSS and QNPHSSNICN did not significantly affect the activity of the enzyme.

**Table 2:** Dipeptidyl-peptidase IV inhibitory activity of SPOT- and traditionally-synthesized deca-peptides.

Category ^a^	Position	Sequence	p*I*	% DPP-IV Inhibition	
				SPOT-Synthesized ^b^	Traditionally-Synthesized ^c,d^
High inhibitory activity, strong binding	C1	LPEWVCTTFH	5.24	26	26
	J6	LAHKALCSEK	8.21	27	31
	K6	LKPTPEGDLE	4.14	23	24
High inhibitory activity, moderate/no binding	F5	WCKDDQNPHS	5.21	29	51
	J11	LCSEKLDQWL	4.37	25	25
	K7	IPAVFKIDAL	5.84	25	48
	K11	YPSKPDNPGE	4.37	31	25
Low inhibitory activity, strong binding	A1	EQLTKCEVFR	6.23	11	10
	A8	VFRELKDLKG	8.58	14	3
	B6	GYGGVSLPEW	4.00	17	8
	C9	FHTSGYDTQA	5.08	12	2
Low inhibitory activity, moderate/no binding	C11	TSGYDTQAIV	3.80	14	−1
	D4	DTQAIVQNND	3.56	14	2
	F6	CKDDQNPHSS	5.21	15	−3
	F10	QNPHSSNICN	6.73	8	2

^a^ High inhibitory activity refers to peptides able to cause more than 20% decrease in DPP-IV activity whereas strong binding refers to peptide spots with a relative luminescence intensity >4000; ^b^ Values shown are averages of four determinations obtained from two separate inhibition experiments; ^c^ Values shown are averages of three determinations; ^d^ Assay performed using 250 µM of peptides (final assay concentration).

### 2.4. Modes of Inhibition of Deca-Peptides

**Figure 5:**
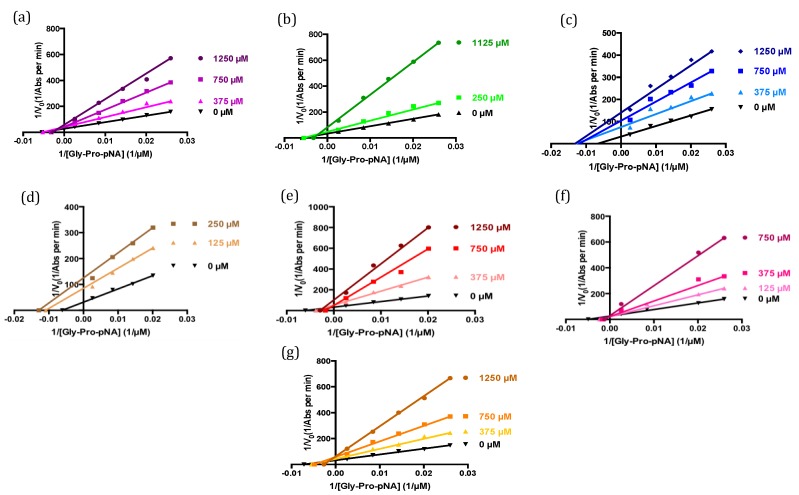
Lineweaver-Burk plots of DPP-IV activity in the absence and presence of peptides. (**a**) LPEWVCTTFH; (**b**) LAHKALCSEK; (**c**) LKPTPEGDLE; (**d**) WCKDDQNPHS; (**e**) LCSEKLDQWL; (**f**) IPAVFKIDAL; (**g**) YPSKPDNPGE.

The deca-peptides shown to cause the greatest inhibition of DPP-IV activity were further studied to determine their inhibition constant (*K*_i_) and identify their modes of action on the DPP-IV enzyme. The sequences LPEWVCTTFH, LAHKALCSEK, IPAVFKIDAL and YPSKPDNPGE were found to inhibit DPP-IV in a competitive manner, whereas WCKDDQNPHS behaved as an un-competitive inhibitor and the decamers LKPTPEGDLE and LCSEKLDQWL exhibited mixed inhibition (Table 3 and Figure 5). The α-lactalbumin-derived peptide WCKDDQNPHS and the β-lactoglobulin-derived sequence IPAVFKIDAL, with α*K*_i_ and *K*_i_ values of 76 and 156 µM, respectively, were shown to have the lowest inhibition constants. Conversely, the deca-peptide LKPTPEGDLE was found to be the least potent inhibitor (*K*_i_ value = 729 µM) (Table 3).

**Table 3:** Inhibition constant (*K*_i_) and mode of inhibition of traditionally synthesized deca-peptides. ^a^ The *K*_i_ (or α*K*_i_ in the case of un-competitive inhibition) values are reported as the averages and standard errors from triplicate determinations.

Sequence	*K*_i_ (µM) ^a^	Mode of Inhibition
LPEWVCTTFH	300 ± 16	Competitive
LAHKALCSEK	217 ± 9	Competitive
LKPTPEGDLE	729 ± 128	Mixed
WCKDDQNPHS	76 ± 5	Un-competitive
LCSEKLDQWL	217 ± 15	Mixed
IPAVFKIDAL	156 ± 11	Competitive
YPSKPDNPGE	262 ± 17	Competitive

## 3. Discussion

The DPP-IV inhibitory activity of a peptide has been suggested to be influenced by a number of factors, including its length, charge and amino acid composition [27]. However, to date, it is still unclear which of these structural or compositional characteristics play a predominant role. In the present study, the net charge of the peptides did not seem to have a predictable effect on their potency nor their ability to bind to the enzyme. Peptides with similar isoelectric point (p*I*), such as LPEWVCTTFH and CKDDQNPHSS, were found to display significantly different DPP-IV inhibitory activity and binding (Table 2 and Figure 2). Similarly, since all sequences investigated in this study were composed of 10 amino acids, the length of the peptides could not be considered a factor influencing their potency and binding behavior. Therefore, the specific amino acid sequences of the peptides, rather than other structural properties, appear to be the predominant element determining their ability to inhibit DPP-IV.

The interactions between an enzyme and ligands that may be potential inhibitors can be investigated by binding and inhibition assays. In the present study, these two approaches were used to study the interaction between the enzyme DPP-IV and deca-peptides derived from α-lactalbumin, a protein known to be a precursor of DPP-IV inhibitory peptides [14,15]. Even though some peptides showed both binding to and inhibitory activity against DPP-IV, overall the results obtained from these two assays were not found to be correlated. This discrepancy may be explained by a number of factors including the fact that binding of the α-lactalbumin-derived peptides to DPP-IV may occur at different domains of the enzyme. Therefore, peptides that showed strong binding but low DPP-IV inhibitory activity, such as EQLTKCEVFR, VFRELKDLKG, GYGGVSLPEW and FHTSGYDTQA, may bind outside the enzyme’s catalytic center without affecting its activity. Interestingly, some of the most potent deca-peptides, including WCKDDQNPHS, LCSEKLDQWL, IPAVFKIDAL and YPSKPDNPGE (Table 2), displayed no or only moderate interaction with DPP-IV based on the binding assay (Figure 2). Investigation on the mode of inhibition of these peptides revealed that WCKDDQNPHS and LCSEKLDQWL exhibited an un-competitive and a mixed mode of action, respectively, suggesting that they may affect enzymatic activity by binding not to the enzyme itself, but rather to the enzyme-substrate complex [28]. The deca-peptides IPAVFKIDAL and YPSKPDNPGE, on the other hand, were found to act as competitive inhibitors and therefore would be expected to interact with DPP-IV since they reduce the enzyme’s activity by binding to and blocking its active site (Table 3 and Figure 5). Since the binding assay is performed directly on the bound peptides, the peptide density in the spots may limit their accessibility to the enzyme. It is also possible that the peptides are able to interact with DPP-IV, but that the binding leads to a change in the enzyme’s conformational structure preventing its recognition by the primary antibody and therefore its detection. Moreover, because each peptide on the array is synthesized with a different yield, depending on the amino acid sequence, hydrophobicity and conformation [29], peptides able to interact with DPP-IV, but that are present in lower concentrations on the membrane, may show no or only moderate binding to the enzyme. Hence, information on the ability of a peptide to bind to DPP-IV cannot be used to predict its ability to inhibit the enzyme.

The SPOT- and traditionally-synthesized decamers showed a consistent trend in their DPP-IV inhibitory activity, thus confirming that peptide array technology can be used to identify DPP-IV inhibitory peptides. Since the yield and purity of peptides on the array are unknown, this approach is only semi-quantitative [25], allowing the comparison of the relative potency of peptides, but not the determination of their absolute DPP-IV inhibitory activity. Nevertheless, peptide arrays could be useful tools to screen a large number of peptides for their DPP-IV inhibitory activity and to identify in the primary sequence of a protein the areas that can inhibit the enzyme. By then exploring where in the tertiary structure of the protein these areas are located, it would be possible to know whether the active peptides are exposed on the surface of the molecule, and thus prone to be cleaved by treatment with proteolytic enzymes, or buried within the interior of the molecule and likely needing to be exposed prior to being released from the protein. Ultimately, this knowledge could help identify the best treatments to generate DPP-IV inhibitory peptides from dietary proteins.

## 4. Experimental Section

### 4.1. Materials

Recombinant human dipeptidyl-peptidase IV (DPP-IV, EC 3.4.14.5, expressed in *HEK293* human cells, >95% purity, activity >5500 pmoles Gly–Pro–7-amido-4-methylcoumarin/min/µg) was purchased from Creative BioMart (Shirley, NY, USA) while polyclonal goat DPP-IV antibody and polyclonal donkey anti-goat secondary antibody conjugated to horseradish peroxidase were obtained from R&D Systems (Minneapolis, MN, USA). Pierce™ ECL western blotting substrate and protein-free (TBS) blocking buffer were from Thermo Scientific (Rockford, IL, USA). Gly–Pro–*p*-nitroanilide (H-Gly–Pro–*p-*NA∙HCl) was purchased from Bachem Americas (Torrance, CA, USA). Synthesized peptides (≥95% purity) were prepared and purified by GL Biochem (Shanghai) Ltd. (Shanghai, China).

### 4.2. Methods

#### 4.2.1. Peptide Array Synthesis

Peptide arrays of deca-peptides spanning the entire α-lactalbumin sequence with a frame shift of one amino acid between successive sequences were prepared by Kinexus Bioinformatics Corporation (Vancouver, BC, Canada). The peptides were synthesized from the *C*-terminal amino acid using the SPOT technology consisting of the stepwise coupling of the Fmoc-protected amino acids and cleavage of the side chain protection groups. The α-lactalbumin-derived peptides were synthesized on trioxatridecanediamine (TOTD) and β-alanine-modified Whatman 540 cellulose membranes for the binding and inhibition assays, respectively. For the inhibition assay, the peptides were synthesized in large dots (5–7 mm in diameter) and the quality of the synthesis was verified by HPLC analysis of quality controls spots included on the membranes.

Along with the 114 deca-peptides derived from the α-lactalbumin sequence, seven additional peptides were included on the arrays (Table S2). Each array was prepared in duplicate.

#### 4.2.2. Probing of the Membrane-Bound Peptide Arrays

The membranes were first blocked with protein-free (Tris-buffered saline (TBS)) blocking buffer containing 0.05% Tween-20 for 4 h at room temperature and then incubated with DPP-IV (10 μg/mL in blocking buffer) overnight at 4 °C. The membranes were washed with TBS (50 mM Tris, 136 mM NaCl, 0.05% Tween-20, pH 8.0) four times for 15 min each, after which they were incubated with the polyclonal goat DPP-IV antibody (0.2 µg/mL) for 8 h at 4 °C. The membranes were once again washed with TBS four times for 15 min each prior to being incubated with anti-goat secondary antibody conjugated to horseradish peroxidase (0.1 µg/mL) overnight at 4 °C. Finally, the membranes were washed four more times for 15 min each; then a thin layer of chemiluminescence detection mixture, prepared according to the manufacturer’s instructions, was applied for 1 min and the luminescence generated from the spots on the membranes with bound DPP-IV was measured using a ChemiDoc™ MP system (Bio-Rad Laboratories (Canada) Ltd., Mississauga, ON, Canada).

#### 4.2.3. DPP-IV Inhibition Assay on SPOT-Synthesized Peptides

In order to determine the effect of the SPOT-synthesized peptides on the activity of the DPP-IV enzyme, the peptides were first cleaved from the cellulose support by incubating the membranes with gaseous ammonia overnight. Peptide spots (5 mm in diameter) were then punched out using a single-hole punch and transferred into microcentrifuge tubes containing 150 µL of acetonitrile (50% in 100 mM Tris–HCl buffer pH 8.0). The peptide solution (50 µL) was pipetted into a 96-well microplate with 35 µL of the chromogenic substrate Gly–Pro–*p*-NA (0.29 mM in 100 mM Tris–HCl buffer pH 8.0). The mixture was pre-incubated for 10 min at 37 °C, whereupon 15 µL of DPP-IV (25 mU/mL) was added and the enzymatic reaction was carried out for 30 min at 37 °C. The reaction was then terminated by addition of 100 µL of 1 M sodium acetate buffer pH 4.0 and the absorbance of the released *p*-nitroaniline was measured at 405 nm using a Tecan Infinite^®^ 200 Pro microplate reader (ESBE Scientific, Markham, ON, Canada). Positive and negative controls (DPP-IV activity with no inhibitor and no DPP-IV activity, respectively) were prepared using punched peptide-free cellulose spots from the same membrane used for peptide synthesis, and adding Tris-HCl buffer in place of the sample and in place of the sample and enzyme solution, respectively.

One unit of DPP-IV was defined as the concentration of enzyme that releases 1 µmol per min of *p*-nitroaniline from Gly–Pro–*p*-NA (100 µM) at 37 °C and pH 8.0.

#### 4.2.4. DPP-IV Inhibition Assay on Traditionally-Synthesized Peptides

A number of SPOT-synthesized peptides that either showed no/low DPP-IV inhibitory activity or that were found to be potent inhibitors were obtained by the traditional solid phase peptide synthesis to confirm their effect on the enzyme.

The peptides were dissolved in a mixture of acetonitrile and H_2_O and further diluted in 100 mM Tris-HCl pH 8.0 buffer to the desired concentrations. In a 96-well microplate, 25 µL of sample were pre-incubated with 25 µL of 100 mM Tris-HCl pH 8.0 buffer and 35 µL of Gly–Pro–*p*-NA (0.29 mM in 100 mM Tris-HCl buffer pH 8.0) at 37 °C for 10 min. The enzymatic reaction was started by addition of 15 µL of DPP-IV solution (25 mU/mL) and carried out at 37 °C for 30 min. Sodium acetate buffer (1 M, pH 4.0, 100 µL) was added to stop the reaction and the absorbance of the released *p*-nitroaniline was measure at 405 nm using a Tecan Infinite^®^ 200 Pro microplate reader. The positive and negative controls were prepared by using Tris-HCl buffer (100 mM, pH 8.0) in place of the sample and in place of the sample and enzyme solution, respectively.

#### 4.2.5. Determination of the Mode of Inhibition and the Inhibition Constant (*K*_i_)

The traditionally-synthesized peptides showing the highest inhibitory activity against DPP-IV were further studied for their mode of action on the enzyme as previously described [15] with the following modifications: the substrate (25 µL) was combined with 100 mM Tris-HCl buffer pH 8.0 (35 µL) and the sample (25 µL) and pre-incubated for 10 min at 37 °C, after which the enzymatic reaction was started by addition of the DPP-IV enzyme (15 µL of a 25 mU/mL solution).

The mode of inhibition and *K*_i_ values of the inhibitory peptides were determined by fitting the initial velocity *versus* substrate concentration data to nonlinear regression models using the GraphPad Prism software (version 6, GraphPad Software, San Diego, CA, USA). The *K*_i_ values are expressed as final assay concentrations.

## 5. Conclusions

Findings from this study showed that peptide arrays on cellulose support can be used to investigate protein-derived peptides for their interaction with the enzyme DPP-IV. By allowing the parallel synthesis of a large number of peptides in small amounts, this method enables the screening of entire protein sequences for active peptides in a timely and cost effective manner.

Since results obtained using peptide array technology are only semi-quantitative, any information gained must be confirmed by other methods. Nevertheless, peptide arrays are useful screening tools for investigating biologically active peptides and could be used to facilitate structure-function studies, and complement or support the traditional methods currently used to identify peptides with DPP-IV inhibitory activity.

## Supplementary Files

Supplementary File 1
