# Supplementary material for: Peptide Array on Cellulose Support—A Screening Tool to Identify Peptides with Dipeptidyl-Peptidase IV Inhibitory Activity within the Sequence of α-Lactalbumin
Source: Int J Mol Sci. 2014 Nov 13;15(11):20846–58. doi: 10.3390/ijms151120846 (PMC4264199; doi:10.3390/ijms151120846)
Supplement: Supplementary File 1 [file ijms-15-20846-s001.pdf]

Supplementary Information

Table S1. Sequences of 114 α-lactalbumin-derived deca-peptides and their position on the arrays. Seven additional peptide sequences not derived from α-lactalbumin are shown in red font.

Position	Peptide Sequence	Position	Peptide Sequence
A1	EQLTKCEVFR	G1	PHSSNICNIS
A2	QLTKCEVFRE	G2	HSSNICNISC
A3	LTKCEVFREL	G3	SSNICNISCD
A4	TKCEVFRELK	G4	SNICNISCDK
A5	KCEVFRELKD	G5	NICNISCDKF
A6	CEVFRELKDL	G6	ICNISCDKFL
A7	EVFRELKDLK	G7	CNISCDKFLD
A8	VFRELKDLKG	G8	NISCDKFLDD
A9	FRELKDLKGY	G9	ISCDKFLDDD
A10	RELKDLKGYG	G10	SCDKFLDDDL
A11	ELKDLKGYGG	G11	CDKFLDDDLT
B1	LKDLKGYGGV	H1	DKFLDDDLTD
B2	KDLKGYGGVS	H2	KFLDDDLTDD
B3	DLKGYGGVSL	H3	FLDDDLTDDI
B4	LKGYGGVSLP	H4	LDDDLTDDIM
B5	KGYGGVSLPE	H5	DDDLTDDIMC
B6	GYGGVSLPEW	H6	DDLTDDIMCV
B7	YGGVSLPEWV	H7	DLTDDIMCVK
B8	GGVSLPEWVC	H8	LTDDIMCVKK
B9	GVSLPEWVCT	H9	TDDIMCVKKI
B10	VSLPEWVCTT	H10	DDIMCVKKIL
B11	SLPEWVCTTF	H11	DIMCVKKILD
C1	LPEWVCTTFH	I1	IMCVKKILDK
C2	PEWVCTTFHT	I2	MCVKKILDKV
C3	EWVCTTFHTS	I3	CVKKILDKVG
C4	WVCTTFHTSG	I4	VKKILDKVGI
C5	VCTTFHTSGY	I5	KKILDKVGIN
C6	CTTFHTSGYD	I6	KILDKVGINY
C7	TTFHTSGYDT	I7	ILDKVGINYW
C8	TFHTSGYDTQ	I8	LDKVGINYWL
C9	FHTSGYDTQA	I9	DKVGINYWLA
C10	HTSGYDTQAI	I10	KVGINYWLAH
C11	TSGYDTQAIV	I11	VGINYWLAHK
D1	SGYDTQAIVQ	J1	GINYWLAHKA
D2	GYDTQAIVQN	J2	INYWLAHKAL
D3	YDTQAIVQNN	J3	NYWLAHKALC
D4	DTQAIVQNND	J4	YWLAHKALCS
D5	TQAIVQNNDS	J5	WLAHKALCSE
D6	QAIVQNNDST	J6	LAHKALCSEK

Table S1. *Cont.*

Position	Peptide Sequence	Position	Peptide Sequence
D7	AIVQNNDSTE	J7	AHKALCSEKL
D8	IVQNNDSTEY	J8	HKALCSEKLD
D9	VQNNDSTEYG	J9	KALCSEKLDQ
D10	QNNDSTEYGL	J10	ALCSEKLDQW
D11	NNDSTEYGLF	J11	LCSEKLDQWL
E1	NDSTEYGLFQ	K1	CSEKLDQWLC
E2	DSTEYGLFQI	K2	SEKLDQWLCE
E3	STEYGLFQIN	K3	EKLDQWLCEK
E4	TEYGLFQINN	K4	KLDQWLCEKL
E5	EYGLFQINNK	K5	IVTQTMKGLD
E6	YGLFQINNKI	K6	LKPTPEGDLE
E7	GLFQINNKIW	K7	IPAVFKIDAL
E8	LFQINNKIWC	K8	HSQGTFTSDY
E9	FQINNKIWCK	K9	YAEGTFISDY
E10	QINNKIWCKD	K10	HAEGTFTSDY
E11	INNKIWCKDD	K11	YPSKPDNPGE
F1	NNKIWCKDDQ		
F2	NKIWCKDDQN		
F3	KIWCKDDQNP		
F4	IWCKDDQNPH		
F5	WCKDDQNPHS		
F6	CKDDQNPHSS		
F7	KDDQNPHSSN		
F8	DDQNPHSSNI		
F9	DQNPHSSNIC		
F10	QNPHSSNICN		
F11	NPHSSNICNI		

Table S2. Characteristics of the seven non-α-lactalbumin-derived deca-peptides included on the arrays. β-lg, beta-lactoglobulin; GIP, glucose-dependent insulinotropic polypeptide; GLP-1, glucagon-like peptide-1; NPY, neuropeptide Y.

Sequence	Origin	Reported Relationship to DPP-IV	Reference
IVTQTMKGLD	β-lg$_{(2–11)}$	Weak inhibitor	[15]
LKPTPEGDLE	β-lg$_{(46–55)}$	Unknown. The ennea- and dodeca-peptides LKPTPEGDL and LKPTPEGDLEIL are inhibitors	[15]
IPAVFKIDAL	β-lg$_{(78–87)}$	Unknown. The ennea-peptide IPAVFKIDA is an inhibitor	[15]
HSQGTFTSDY	Glucagon$_{(1–10)}$	Inhibitor	[27]
YAEGTFISDY	GIP$_{(1–10)}$	*N*-terminal of natural substrate	[30]
HAEGTFTSDY	GLP-1$_{(1–10)}$	*N*-terminal of natural substrate	[30]
YPSKPDNPGE	NPY$_{(1–10)}$	*N*-terminal of natural substrate	[30]
